# Evaluation of the prognostic value of all four HER family receptors in patients with metastatic breast cancer treated with trastuzumab: A Hellenic Cooperative Oncology Group (HeCOG) study

**DOI:** 10.1371/journal.pone.0207707

**Published:** 2018-12-06

**Authors:** Angelos Koutras, Georgios Lazaridis, Georgia-Angeliki Koliou, George Kouvatseas, Christos Christodoulou, Dimitrios Pectasides, Vassiliki Kotoula, Anna Batistatou, Mattheos Bobos, Eleftheria Tsolaki, Kyriaki Papadopoulou, George Pentheroudakis, Pavlos Papakostas, Stavroula Pervana, Kalliopi Petraki, Sofia Chrisafi, Evangelia Razis, Amanda Psyrri, Dimitrios Bafaloukos, Konstantine T. Kalogeras, Haralambos P. Kalofonos, George Fountzilas

**Affiliations:** 1 Division of Oncology, Department of Medicine, University Hospital, University of Patras Medical School, Patras, Greece; 2 Department of Medical Oncology, Papageorgiou Hospital, Aristotle University of Thessaloniki, School of Health Sciences, Faculty of Medicine, Thessaloniki, Greece; 3 Section of Biostatistics, Hellenic Cooperative Oncology Group, Data Office, Athens, Greece; 4 Health Data Specialists Ltd, Athens, Greece; 5 Second Department of Medical Oncology, Metropolitan Hospital, Piraeus, Greece; 6 Oncology Section, Second Department of Internal Medicine, Hippokration Hospital, Athens, Greece; 7 Department of Pathology, Aristotle University of Thessaloniki, School of Health Sciences, Faculty of Medicine, Thessaloniki, Greece; 8 Laboratory of Molecular Oncology, Hellenic Foundation for Cancer Research/Aristotle University of Thessaloniki, Thessaloniki, Greece; 9 Department of Pathology, Ioannina University Hospital, Ioannina, Greece; 10 Department of Medical Oncology, Ioannina University Hospital, Ioannina, Greece; 11 Oncology Unit, Hippokration Hospital, Athens, Greece; 12 Department of Pathology, Papageorgiou Hospital, Thessaloniki, Greece; 13 Pathology Department, Metropolitan Hospital, Piraeus, Greece; 14 Third Department of Medical Oncology, Hygeia Hospital, Athens, Greece; 15 Division of Oncology, Second Department of Internal Medicine, Attikon University Hospital, Athens, Greece; 16 First Department of Medical Oncology, Metropolitan Hospital, Piraeus, Greece; 17 Translational Research Section, Hellenic Cooperative Oncology Group, Athens, Greece; 18 Aristotle University of Thessaloniki, Thessaloniki, Greece; University of South Alabama Mitchell Cancer Institute, UNITED STATES

## Abstract

In the current study, we performed a complete analysis, with four different methods, of all four HER family receptors, in a series of patients with metastatic breast cancer treated with trastuzumab-based regimens and evaluated their prognostic value. Formalin-fixed paraffin-embedded tumor tissue samples were collected from 227 patients, considered to be HER2-positive when assessed at the local laboratories. We evaluated gene amplification, copy number variations (CNVs), mRNA and protein expression of all four HER family members. In addition, our analysis included the evaluation of several other factors by immunohistochemistry (IHC), such as pHER2^Tyr1221/1222^, pHER2^Tyr877^ and PTEN. Central review of HER2 status by IHC and fluorescence in situ hybridization revealed that of the 227 patients, only 139 (61.2%) were truly HER2-positive. Regarding the 191 patients treated with trastuzumab as first-line therapy, median time to progression (TTP) was 15.3 and 10.4 months for HER2-positive and HER2-negative participants, respectively, whereas median survival was 50.4 and 38.1 months, respectively. In HER2-positive patients, high HER3 mRNA expression was of favorable prognostic significance for TTP and survival (HR = 0.43, 95% CI 0.21–0.88, Wald’s p = 0.022 and HR = 0.43, 95% CI 0.21–0.88, p = 0.021, respectively), while *EGFR* copy gain and EGFR protein expression were associated with higher risk for disease progression in HER2-negative patients (HR = 3.53, 95% CI 1.19–10.50, p = 0.023 and HR = 3.37, 95% CI 1.12–10.17, p = 0.031, respectively). Positive HER3 protein expression was a favorable factor for TTP in HER2-negative patients (HR = 0.43, 95% CI 0.22–0.84, p = 0.014). In the multivariate analysis, only *EGFR* copy gain retained its prognostic significance for TTP in the HER2-negative population (HR = 3.96, 95% CI 1.29–12.16, p = 0.016), while high HER3 mRNA expression retained its favorable prognostic significance for TTP in the HER2-positive subgroup (HR = 0.47, 95% CI 0.23–0.99, p = 0.048). The present study suggests that *EGFR* copy gain represents a negative prognostic factor for TTP in HER2-negative patients with metastatic breast cancer treated with trastuzumab. In addition, high HER3 mRNA expression appears to be of favorable prognostic significance for TTP in HER2-positive patients. Given the small number of patients included in the current analysis and the retrospective nature of the study, our findings should be validated in larger cohorts.

## Background

Metastatic breast cancer (MBC) is an incurable disease. Systemic treatment generally has a palliative role. However, newer therapeutic agents may prolong the survival of patients with advanced disease [1]. The cell surface, human epidermal growth factor receptor 2 (HER2) is overexpressed in approximately 20% of breast tumors (usually due to HER2 gene amplification) and is associated with an aggressive course of the disease and unfavorable clinical outcome [2]. Trastuzumab, a recombinant humanized monoclonal antibody that selectively targets the extracellular domain of the HER2 receptor, was found to significantly prolong overall survival (OS) of patients with metastatic HER2 over-expressing and/or amplified breast cancer [3]. However, a substantial percentage of patients with HER2-positive MBC receiving trastuzumab will not respond, as a result of inherent resistance. Furthermore, in the majority of responding patients, acquired resistance to treatment is expected to eventually develop. Therefore, the identification of those patients who may derive benefit from this treatment is of major importance in the management of women with HER2-positive MBC. However, apart from HER2 status, no other conclusive biomarkers have been identified, which would further predict those HER2-positive patients that are most likely to respond to trastuzumab treatment.

The HER family includes four members, epidermal growth factor receptor (EGFR), HER2, HER3 and HER4 [4]. The evaluation of all HER members as a whole may be important, given the significance of dimerization among lateral signaling partners [5]. Even though HER2 status can be assessed via different techniques, immunohistochemistry (IHC) remains the most widely used assay for HER2 determination in daily practice. Equivocal cases, (2+) by IHC, necessitate the evaluation of HER2 gene amplification using fluorescence in situ hybridization (FISH) [6]. Quantitative reverse transcription-polymerase chain reaction (qRT-PCR) constitutes an alternative approach for the assessment of HER2 status [7], although its use has not routinely been adopted thus far.

The present retrospective study included samples from 227 patients with presumed HER2-positive MBC who were treated with trastuzumab-based regimens, after local evaluation for HER2 by IHC and FISH (when needed). In the current study, we performed a thorough analysis of all four HER family members evaluating gene amplification, copy number variations (CNVs), transcriptional profiling (mRNA expression) and protein expression of the receptors. In addition, our analysis included the evaluation of several other factors by IHC, such as pHER2^Tyr1221/1222^, pHER2^Tyr877^ and PTEN. Moreover, we investigated the potential associations of all of the above-mentioned factors with each other, when appropriate, and with the outcome of the trastuzumab-treated patients.

## Methods

### Patients

The medical records of all patients with MBC treated with trastuzumab-based regimens, between December 1998 and January 2010, were retrospectively reviewed, as previously described in detail [8–12]. Eligibility criteria for the study were a: histologically confirmed MBC; b: adequacy of clinical data on patient’s history, demographics, tumor characteristics, treatment details (drug dosages, schedule of administration, serious toxicities) and clinical outcome; c: availability of adequate tumor tissue for biological marker evaluation; and d: trastuzumab-based treatment for metastatic disease [8–12]. The translational research protocol was approved by the Bioethics Committee of the Aristotle University of Thessaloniki School of Medicine (Protocol #4283; Jan 14, 2008) under the general title “Investigation of major mechanisms of resistance to treatment with trastuzumab in patients with metastatic breast cancer”. All patients included in the study after 2005 provided written informed consent for the provision of biological material for future research studies before receiving any treatment. Waiver of consent was obtained from the Bioethics Committee for patients treated before 2005.

## Tumor tissue material

**Fig 1:**
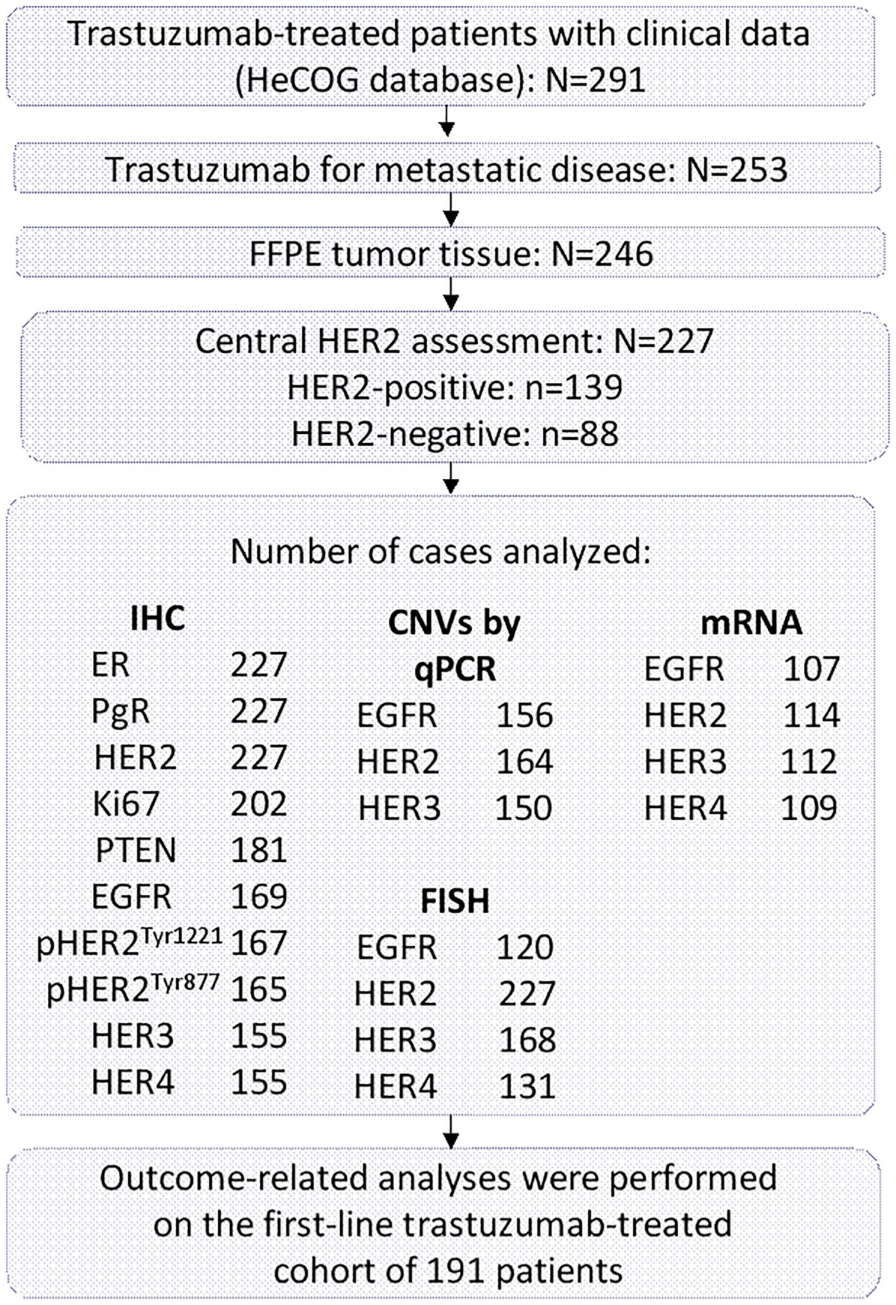
REMARK diagram.

Formalin-fixed paraffin-embedded (FFPE) tumor tissue samples were retrospectively collected from 246 breast cancer patients treated with trastuzumab-based regimens in the metastatic setting, as previously described in detail [8–12]. Nineteen cases were excluded for inadequate FFPE tumor tissue, thus decreasing the number of eligible/evaluable patients to 227. All carcinomas had initially been diagnosed as HER2-positive and thereafter all patients had been treated with trastuzumab. Due to the long period of patient recruitment during which estrogen receptor (ER), progesterone receptor (PgR) and HER2 guidelines for breast cancer typing and patient stratification for trastuzumab treatment were repeatedly modified, all tumors were re-evaluated centrally for these basic breast cancer typing parameters, according to the ASCO/CAP guidelines [13]. A REMARK diagram for the translational research studies included in this investigation is provided in Fig 1.

## Tissue microarrays (TMAs)

Hematoxylin-eosin stained sections from the tissue blocks were reviewed by one pathologist (M.B.). Seventeen TMA blocks were constructed from the 227 available cases using a manual tissue microarrayer (Beecher Instruments, Sun Prairie, WI), as previously described [8]. For the construction of the TMA blocks, 2 core samples (1.5 mm in diameter) were obtained from representative regions of each tumor in the donor blocks. All IHC and FISH markers were assessed on TMA sections. Cases not represented, with damaged or inadequate cores on the TMA sections were re-cut from the original blocks if still available and these sections were used for protein and gene analyses, as previously described in detail [8–11].

## Immunohistochemistry (IHC)

Serial 2.5 μm thick TMA sections or whole tissue sections were stained for ER (clone 6F11, dilution 1:70, Leica Biosystems, Newcastle, UK), PgR (clone 1A6, dilution 1:70, Leica Biosystems), HER2 (polyclonal Ab, code A0485, dilution 1:200, Dako, Glostrup, Denmark), Ki67 (clone MIB-1, code M7240, dilution 1:70, Dako) and PTEN (clone 6H2.1, Dako), as previously described in detail [14]. Sections were also stained with antibodies against EGFR (clone 31G7, code 28–005, Invitrogen Corporation, Frederick MD, dilution 1:50), HER3 (clone SGP1, Thermo Fisher Scientific, Fremont, CA, dilution 1:80), HER4 (clone 83B10, Cell Signaling Technology, Danvers, MA, dilution 1:400), pHER2^Tyr1221/1222^ (clone 6B12, code 2243L, Cell Signaling Technology, dilution 1:400) and pHER2^Tyr877^ (code 2241S, Cell Signaling Technology, dilution 1:1000). The Bond-Max autostainer was used for antigen unmasking (Leica Microsystems, Wetzlar, Germany) and the i6000 automated staining instrument (BioGenex, San Ramon, CA) for the final steps of the IHC method. Diaminobenzidine (DAB, Dako) was used as a chromogen and Mayer’s hematoxylin (BioGenex) as a counterstain.

All sections were stained in one run for each antibody and were evaluated by pathologists experienced in breast cancer (A.B., M.B., S.P., K.P.) and blinded as to the patient’s clinical characteristics and survival data. Positive controls were used for all antibodies from known positive breast cancer cases, while negative controls were obtained by omitting the primary antibody, as previously described in detail [8–11].

## Interpretation of the IHC results

Briefly, ER and PgR were considered positive if staining was present in ≥1% of tumor cell nuclei [15]. HER2 protein expression was scored in a scale from 0 to 3+, the latter corresponding to uniform, intense membranous staining in >30% of invasive tumor cells [13]. For Ki67, the expression was defined as low (<20%) or high (≥20%) based on the percentage of stained/unstained nuclei from the tumor areas [16]. This cut-off was used for classifying Luminal A and B tumors in the HER2-negative subgroup (as assessed by central testing). PTEN protein expression (cytoplasmic, nuclear or both) was evaluated according to a staining intensity scale from 0 (negative, no staining) to 3 (intense staining). Tumors with PTEN scores of 0 or 1 were considered as having PTEN loss [8].

**Fig 2:**
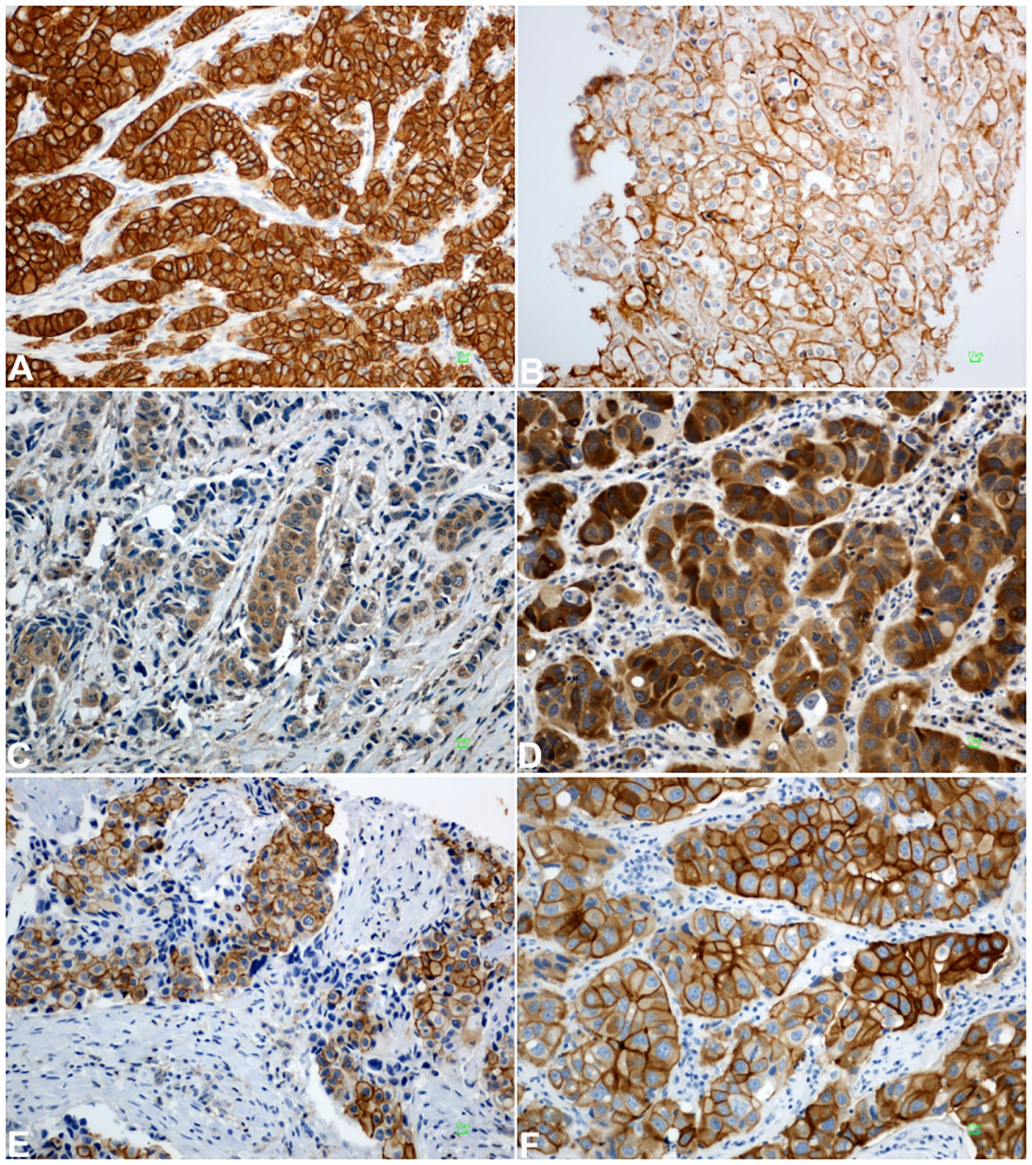
Representative staining images of proteins studied in invasive breast carcinomas. **A**. EGFR expression detected as moderate (2+) and strong (3+) membranous staining in neoplastic cells. **B**. Uniform, intense HER2 membranous staining (3+) in the majority of tumor cells. **C**. Moderate cytoplasmic HER3 expression in tumor cells. **D**. Moderate (2+) and strong (3+) cytoplasmic HER4 expression. **E**. Cytoplasmic and membranous expression of pHER2^Tyr1221/1222^. **F**. Strong (3+) membranous and moderate (2+) cytoplasmic staining of tumor cells for pHER2^Tyr877^. Bar: 10 μm. Magnification: x200.

EGFR was considered positive when ≥1% of the tumor cells had membranous staining above the background level, while the intensity of the EGFR reactivity was scored as +1 (mild), +2 (moderate) and +3 (strong) [17]. For HER3, HER4, pHER2^Tyr1221/1222^ and pHER2^Tyr877^ the staining intensity was scored as +1 (mild), +2 (moderate) and +3 (strong). The tumor was considered to be positive if at least 1+ intensity was observed in ≥10% of neoplastic cells [18]. Representative staining images of all evaluated HER family proteins are shown in Fig 2.

If one of the tissue cores was lost or damaged the overall score was determined from the remaining core. When whole tissue sections were used, the entire tumor area was evaluated.

## Fluorescence in situ hybridization (FISH)

Tissue microarray sections or whole tissue sections (5 μm thick) were used for FISH analysis using the ZytoLight SPEC *HER2/TOP2A/CEN17* triple color probe kit for *HER2* (code Z-2093, ZytoVision, Bremerhaven, Germany). FISH was performed according to the manufacturer’s protocol with minor modifications in all cases, not only the HER2 IHC 2+ cases. The ZytoVision probes used for *EGFR*, *HER3* and *HER4* FISH analysis, were *EGFR/CEN7* (code Z-2033), *HER3/CEN12* (code Z-2056) and *HER4/CEN2* (code Z-2057), respectively. Digital images were constructed using specifically developed software for cytogenetics (XCyto-Gen, ALPHELYS, Plaisir, France), as previously described in detail [8–11, 19].

## FISH evaluation

Processed sections were considered eligible for *HER2* FISH evaluation according to the ASCO/CAP criteria [13]. For the evaluation of the *HER2* gene status, non-overlapping nuclei from the invasive part of the tumor were randomly selected, according to morphological criteria using DAPI staining, and scored. Twenty tumor nuclei were counted according to Press et al [20]. The *HER2* gene was considered to be amplified when the *HER2*/*CEP17* ratio was >2.2 [13], or the mean *HER2* copy number was >6 [21]. In cases with values at or near the cut-off (1.8–2.2), 20–40 additional nuclei were counted and the ratio was recalculated. In cases with ratios that were still borderline, additional FISH assays were performed in whole sections [21]. The data from the evaluation of *TOP2A* gene status were neither analyzed nor presented in the present manuscript.

**Fig 3:**
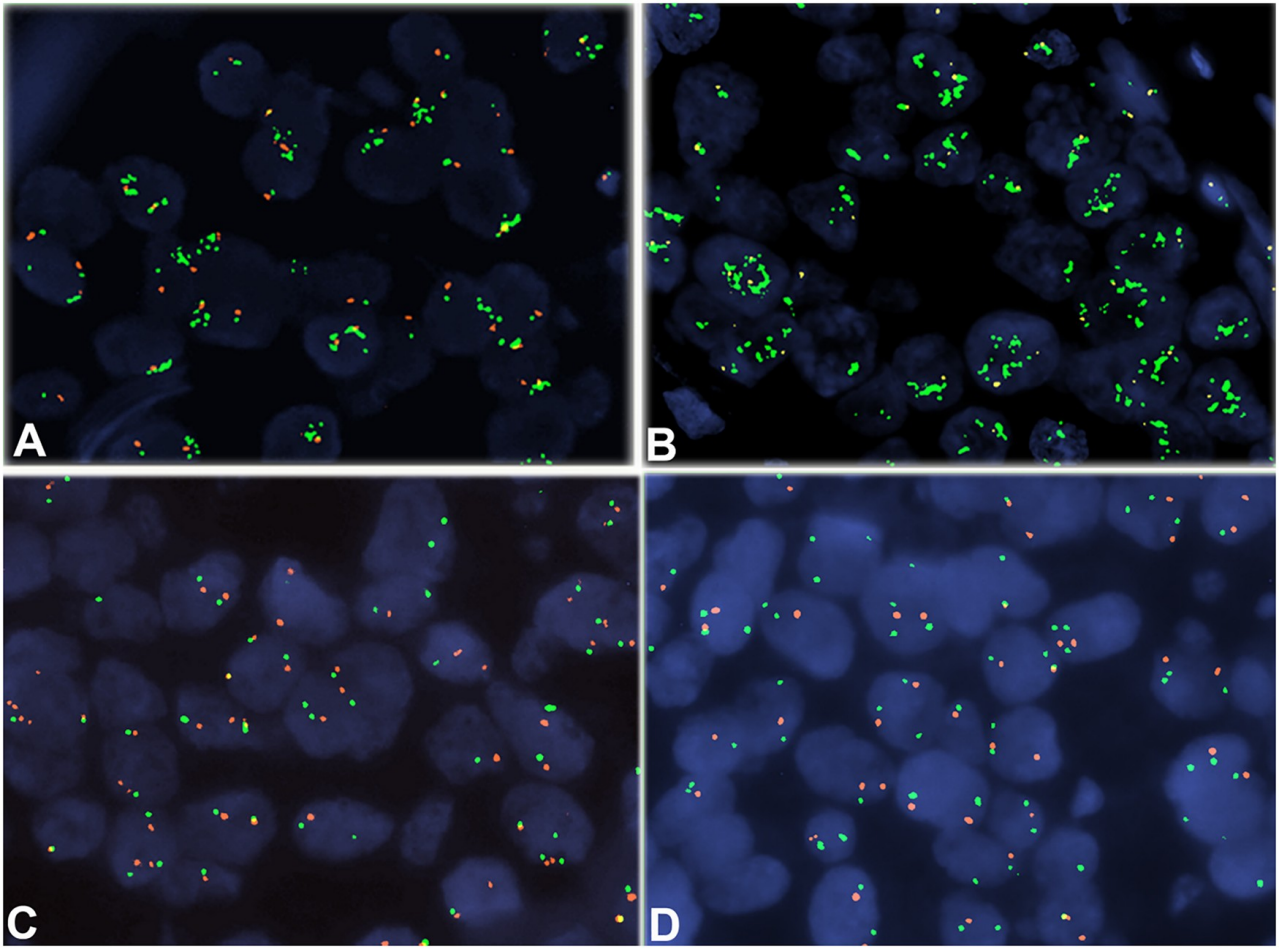
Representative FISH photomicrographs using gene and centromere specific probes performed on TMA slides. **A**. Amplification of the EGFR gene (green signals; gene probe). **B**. High amplification of the HER2 gene. **C**. Tumor cells with normal HER3 gene status. **D**. HER4 non-amplified invasive breast carcinoma. Magnification: x1000.

*EGFR*, *HER3* and *HER4* gene status was assessed in 60 non-overlapping nuclei from the invasive part of the tumor. Gene amplification and centromere (CEN) copy numbers were evaluated with cut-offs based on signal counts in the nuclei of normal breast epithelium from 20 women that had undergone reduction mastectomy. Because only the specific gene loci and the centromeres were assessed per chromosome, observing >2 centromere signals did not necessarily correspond to polysomy or altered ploidy in terms of altered copies of the entire chromosome. Therefore, these terms were not used. Cut-offs for the amplification of *EGFR*, *HER3* and *HER4* and for an increase in their respective CEN copy numbers were calculated as mean gene signal counts and mean CEN signal counts, plus 3 standard deviations, as previously suggested by Watters et al [22]. Based on this approach, amplification ratio (gene copy number/CEN copy number) cut-offs were >2.08 for *EGFR*, >2.39 for *HER3* and >2.07 for *HER4*. In addition, the three genes were considered to be amplified when >4 average gene copies were found. Representative FISH photomicrographs using gene and centromere specific probes of all evaluated HER family members are shown in Fig 3.

## Dual nucleic acid extraction and DNA analyses for *PIK3CA* mutations and *EGFR*, *HER2* and *HER3* copy number variations (CNVs)

All tumors were histologically evaluated on hematoxylin and eosin (H&E) sections for tumor presence and marked for areas with highest tumor density. Tumor cell content (TCC%) was assessed as the ratio of cancer cell vs. non-cancer cell sections in these areas, which were used for manual macrodissection for DNA/RNA extraction [23]. Manual macrodissection was performed on 10 micron thick unstained sections and processed for dual nucleic acid extraction with silica-coated magnetic beads (Versant Tissue Preparation Reagents, Siemens Healthcare Diagnostics, Tarrytown, NY), according to the manufacturer’s instructions. Based on the abundance of tumor tissue on blocks and the availability of thick sections, extracts were divided into two aliquots for storage at -80°C until use. DNase I was added to one aliquot per sample for removing DNA and ensuring the presence of pure RNA for gene expression analyses, as previously described in detail [12].

Mutation testing for hotspot *PIK3CA* mutations E542K and E545K (coding exon 9) and H1047R (coding exon 20) was accomplished with custom Taqman-MGB-SNP genotyping assays (duplex qPCR for the detection of control DNA and mutant target in the same reaction, as previously described [8].

Copy number (CN) analysis for the *EGFR*, *HER2* and *HER3* genes was implemented with quantitative PCR (qPCR) in 180 DNA samples, with premade CNV assays (Life Technologies/Applied Biosystems, Paisley, UK). For each gene, two genomic regions were targeted in order to increase analysis specificity and sensitivity. Assay ID, Genbank reference, location within gene and amplicon size for the genes analyzed were: *EGFR* on chromosome 7p12 (Hs01426560_cn; NM_005228.3, NM_201282.1, NM_201283.1, NM_201284.1; exon 2; 94 bp / Hs02822119_cn; NM_005228.3, NM_201282.1, NM_201283.1, NM_201284.1; exon 10; 75 bp), *HER2* on chromosome 17q21.1 (Hs01839367_cn; NM_001005862.1, NM_004448.2; exon 13; 98 bp / Hs00709630_cn; NM_001005862.1, NM_004448.2; exon 21; 101 bp); and *HER3* on chromosome 12q13.2 Hs00160884_cn; NM_001005915.1, NM_001982.3; exon 1; 95 bp / Hs02591325_cn; NM_001982.3; exon 13; 104 bp). The method involves duplex reactions; for the target genes TaqMan minor groove binding (MGB) probes, FAM labeled; for the reference gene Taqman VIC-TAMR labeled probes; both assays with unlimited primers. TaqMan Copy Number Reference Assay RNase P was used as endogenous reference. Reactions (10 ul) were run in quadruplicates in an ABI7900HT system in 384-well plates under default conditions. Three peripheral blood DNA samples from non-cancer patients were included in each run as calibrator samples, along with no-template controls. Results were obtained automatically with the CopyCaller Software v2.0 as predicted CN, in comparison to averaged calibrator values upon setting the evaluation threshold at Cp (crossing point) = 32 for the reference RNase P in each reaction. The ΔΔCT method was employed to estimate the CT difference (ΔCT) between target and reference sequence in tumor samples as compared to the corresponding values of the calibrator samples. Based on the Cp = 32 eligibility cut-off, out of 180 samples, 156 were informative for *EGFR*, 164 were informative for *HER2* and 150 for *HER3* copy number assessment (92.8%, 95.0% and 88.9%, respectively).

*EGFR* and *HER3* CNVs were classified as no gain for quadruplicate average CN ≤2.5 and as gain for average CN >2.5. The cut-off 2.5 was chosen arbitrarily in order to exclude DNA replication. *EGFR* and *HER3* CN values were <5. Since *HER2* CN values reached up to 18, a 3-scale classification was applied for this gene, i.e., <2.5 copies: no gain (normal); 2.5–5 copies: low gain; >5 copies: high gain. Both assays per gene had to be informative for the tumor evaluation. In cases with discrepant results between the two assays (no gain and gain for the same tumor), gain was called for that particular gene.

## Relative EGFR, HER2, HER3 and HER4 mRNA expression (qRT-PCR)

cDNA synthesis was applied on 204 RNA samples, with random primers and SuperScript III Reverse Transcriptase (Invitrogen, Paisley, UK; cat. no. 48190011 and 18080044, respectively), according to the manufacturer’s instructions. cDNAs were assessed in duplicate 10 ul reactions in 384-well plates with qRT-PCR in an ABI7900HT system for 45 cycles of amplification (default conditions). The following exon-spanning premade Taqman-MGB assays (Applied Biosystems/Life Technologies) were selected for the transcripts under investigation (data in parentheses refer to assay ID; Genbank reference; amplicon location; size): *EGFR* (Hs00193306_m1; NM_005228.3; exons 20–21; 69 bp), *HER2* (Hs00170433_m1; NM_001005862.1, NM_004448.2; exons 18–19, 15–16; 120 bp), *HER3* (Hs00176538_m1; NM_001005915.1, NM_001982.3; exons 1–2; 62 bp), *HER4* (Hs00171783_m1; NM_001042599.1, NM_005235.2; exons 12–13; 77 bp). A Taqman-MGB expression assay targeting β-glucuronidase (GUSB) mRNA (Hs00939627_m1; NM_000181.3; exons 8–9; 96 bp) was used as the endogenous reference for the assessment of relative quantification. *GUSB* was selected because, among the widely used housekeeping genes, it does not seem to be represented in pseudogenes. In addition, GUSB has been independently identified as one among the best-preserved mRNA targets in FFPE tissues [24, 25]. A commercially available reference RNA derived from multiple transformed cell lines (TaqMan Control Total RNA, cat. no 4307281, Applied Biosystems) was applied in multiple positions in each run as positive control and for inter-run evaluation of PCR assay efficiency. To obtain linear relative quantification (RQ) values, relative expression was assessed as (40-dCT), whereby dCT (or delta cycle threshold, equivalent to Cq in MIQE guidelines) was calculated as (average target CT)–(average GUSB CT) from all eligible measurements under the same reading threshold. Samples were considered eligible for GUSB CT <36 and deltaRQ for each duplicate pair (intra-run variation) of <0.8. Inter-run RQ values for the reference RNA were <1 for both assays.

## Statistical analysis

Follow-up information for all patients was updated in January 2017. Distribution characteristics of all examined biomarkers in HER2-positive and HER2-negative patients were evaluated and presented. The frequencies and corresponding percentages are provided for the categorical variables, while the median and range is presented for the continuous ones. Chi-square or Fisher’s exact (where appropriate) tests were used for group comparisons of categorical data, while Wilcoxon rank-sum tests were performed to detect differences in continuous variables.

Time to progression (TTP) was defined as the time from the initiation of the trastuzumab first-line treatment (with or without concurrent chemotherapy or hormonal therapy) to the date of documented disease progression. Survival was also measured from the initiation of trastuzumab first-line treatment to the date of death. Patients who survived without relapse were censored at the date of their last contact. The prognostic value of mRNA expression was evaluated in terms of TTP and survival, using the 50th percentile (median value) as the optimal cut-off and if this was not significant the upper and lower quartiles were to be examined, as possible thresholds. The cut-offs described earlier in this section of the paper were used for the rest of the examined markers.

The outcome-related analyses focused on HER2-positive and HER2-negative patients (as determined by HER2 central assessment) who received trastuzumab in the first-line of treatment and were performed separately for these two population groups. The associations between factors of interest and progression/mortality rates were evaluated by hazard ratios (HR) estimated with univariate and multivariate Cox proportional hazards models. Kaplan-Meier curves were used for estimating time-to-event distributions, while log-rank tests were performed to assess predefined comparisons. Variables were tested for proportionality using time-dependent covariates and by graphical checks. Some evidence of violation of the proportionality assumption for the variable describing the patients’ disease presentation status was observed and therefore stratified Cox multivariate models were applied with disease presentation status (*de novo* MBC vs. relapsed MBC patients) as the stratification variable.

Model choice was performed using backward selection criteria at p<0.15, including the following clinicopathological parameters in the initial step: menopausal status (postmenopausal vs. premenopausal), ER/PgR status (positive vs. negative), performance status (1–2 vs. 0), number of metastatic sites (≥4 vs. 1–3), as well as one of the markers that was found to be significant in the univariate analysis.

Results of this study are presented according to reporting recommendations for tumor marker prognostic studies [26]. All tests are two-sided at an alpha 5% level of significance. No adjustment for multiple comparisons was performed. Analyses were conducted using the SAS software (version 9.3, SAS Institute Inc., Cary, NC).

## Results

Among the 227 eligible patients with MBC treated with trastuzumab, only 139 (61.2%) were found to have centrally assessed HER2 gene amplification by FISH and/or 3+ HER2 protein overexpression by IHC (Fig 1). Eighty-nine of the HER2-positive patients (64.0%) were luminal-HER2, with the rest (36.0%) being HER2-enriched (Table 1). It is worth mentioning that all 227 cases were classified as HER2-positive locally, when evaluated with IHC (and FISH in some circumstances) and had thus received trastuzumab. Consequently, 88 HER2-negative patients had been treated with trastuzumab-based regimens, possibly due to inexperience of local laboratories with HER2 IHC assessment at that time. Table 1 presents selected patient and disease characteristics of both patient groups at the initiation of trastuzumab treatment. Patients diagnosed with stage IV breast cancer were considered as *de novo* MBC, while patients diagnosed at earlier stages of the disease were characterized as relapsed metastatic breast cancer patients (R-MBC). For the majority of the R-MBC patients (85%) HER2 status was evaluated in primary tumors.

**Table 1:** Selected patient and tumor characteristics (at trastuzumab initiation) according to HER2 status.

	HER2 status		
	**Total**	**Negative**	**Positive**
N	227	88	139
**Age (years)**			
Median (Range)	56.3 (28.4–95)	58.3 (32–79)	54.6 (28.4–95)
	**N (%)**	**N (%)**	**N (%)**
***de novo* MBC**	71 (31.3)	28 (31.8)	43 (30.9)
**R-MBC**	156 (68.7)	60 (68.2)	96 (69.1)
History of neoadjuvant CT*	15 (9.6)	6 (10.0)	9 (9.4)
History of adjuvant CT*	127 (81.4)	50 (83.3)	77 (80.2)
Anthracycline-based CT*	96 (61.5)	31 (51.7)	65 (67.7)
Taxane-containing CT*	54 (34.6)	16 (26.7)	38 (39.6)
CMF-based CT*	73 (46.8)	29 (48.3)	44 (45.8)
History of adjuvant HT*	110 (70.5)	45 (75.0)	65 (67.7)
History of adjuvant RT*	84 (53.8)	33 (55.0)	51 (53.1)
**Number of metastatic sites**			
1–3	206 (90.7)	79 (89.8)	127 (91.4)
≥4	20 (8.8)	8 (9.1)	12 (8.6)
Unknown	1 (0.4)	1 (1.1)	0 (0.0)
**Histological grade**			
I-II	95 (41.9)	40 (45.4)	55 (39.6)
III	117 (51.5)	41 (46.6)	76 (54.6)
Unknown	15 (6.6)	7 (8.0)	8 (5.8)
**Menopausal status**			
Premenopausal	57 (25.1)	21 (23.9)	36 (25.9)
Postmenopausal	168 (74.0)	67 (76.1)	101 (72.7)
Unknown	2 (0.9)	0 (0.0)	2 (1.4)
**Number of trastuzumab lines**			
1	76 (33.5)	32 (36.4)	44 (31.7)
2	54 (23.8)	21 (23.7)	33 (23.7)
3	39 (17.2)	14 (15.9)	25 (18.0)
≥4	58 (25.6)	21 (23.9)	37 (26.6)
**Performance status**			
0	161 (71.0)	61 (69.3)	100 (72.0)
1	52 (22.9)	20 (22.7)	32 (23.0)
2	13 (5.7)	6 (6.8)	7 (5.0)
Unknown	1 (0.4)	1 (1.1)	0 (0.0)
**Subtype classification**			
Luminal A	15 (6.6)	15 (17.0)	0 (0.0)
Luminal B	53 (23.3)	53 (60.2)	0 (0.0)
Luminal-HER2	89 (39.2)	0 (0.0)	89 (64.0)
HER2-Enriched	50 (22.0)	0 (0.0)	50 (36.0)
TNBC	13 (5.7)	13 (14.8)	0 (0.0)
Unknown	7 (3.1)	7 (8.0)	0 (0.0)
**Sites of metastasis**			
Locoregional	76 (33.5)	28 (31.8)	48 (34.5)
Distant	199 (87.7)	77 (87.5)	122 (87.8)
Only locoregional	17 (7.5)	6 (6.8)	11 (7.9)
Only distant	138 (60.8)	54 (61.4)	84 (60.4)
Bones	96 (42.3)	40 (45.5)	56 (40.3)
Nodes	44 (19.4)	15 (17.0)	29 (20.9)
Visceral metastases	150 (66.1)	53 (60.2)	97 (69.8)

HER2, human epidermal growth factor receptor 2; MBC, metastatic breast cancer; R-MBC, relapsed metastatic breast cancer; CT, chemotherapy; HT, hormonal therapy; RT, radiotherapy; CMF, cyclophosphamide/methotrexate/5 fluorouracil; TNBC, triple-negative breast cancer.

*Only for relapsed metastatic breast cancer patients.

Trastuzumab was administered as first-line therapy in 191 cases (84.1%), whereas in the remaining 36 of the 227 evaluable patients (15.9%), trastuzumab was given later in the course of advanced disease. Consequently, these 36 cases were not included in the outcome-related analyses, which were restricted to the population of 191 patients receiving trastuzumab in the first-line setting (125 HER2-positive and 66 HER2-negative cases), while all other analyses were performed in the entire cohort of 227 evaluable patients. It is worthy to note that 5 patients received trastuzumab without concurrent chemotherapy or hormonal therapy, while 8 patients were treated with trastuzumab in the adjuvant and/or neo-adjuvant setting, as well.

Median follow-up for all first-line patients included in the study was 129.9 months (range 0.6–192.6), while HER2-positive and HER2-negative patients who received first-line treatment of trastuzumab were followed-up for a median of 138 (range 0.6–192.6) and 126 (range 1.0–142.9) months, respectively. At the time of the analysis, 56 of the 66 HER2-negative patients (84.8%) treated with trastuzumab in the first-line had died and 55 patients (83.3%) had disease progression. Similarly, 96 of the 125 patients (76.8%) with HER2-positive tumors that were treated with first-line trastuzumab therapy had died and 98 patients (78.4%) had experienced a relapse. In total, 23 of the 191 patients (9 HER2-negative and 14 HER2-positive) treated with trastuzumab in the first-line died within a year since trastuzumab initiation. The median TTP was 15.3 months (range 0.6–192.6) and 10.4 months (range 1.6–136.0) for the HER2-positive and HER2-negative participants that received first-line trastuzumab therapy, respectively. Median survival was 50.4 months (range 0.6–192.6) for HER2-positive patients treated with trastuzumab in the first-line and 38.1 months (range 4.5–142.9) for HER2-negative patients. No significant differences were observed between HER2-positive and HER2-negative patients in terms of TTP and survival (log-rank, p = 0.15 and p = 0.06, respectively).

Table 2 presents the frequency distribution of all the important markers included in this study by category and HER2 status for the total cohort. It is of note that only four out of the 120 (3.3%) evaluable tumors had *EGFR* amplification, 1.8% had *HER3* amplification, while no cases of *HER4* amplification were observed. Three out of the four patients with *EGFR* amplified tumors had normal *EGFR* and *HER3* CN, while all four of them had *HER2* low gain. In addition, three out of the four *EGFR* amplified tumors did not express EGFR protein. Of the three tumors with *HER3* amplification, one tumor presented with normal *EGFR*, *HER2* and *HER3* CN, one tumor had normal *EGFR* and *HER3* CN and *HER2* low gain, while one of the three HER3 amplified tumors was non-informative regarding CN status. Although these numbers are too small for statistical analyses to be performed, the described profiles indicate that the EGFR protein expression in the examined tumors was not related to underlying gene pathology. HER3 protein expression status was available for two of the three tumors with *HER3* amplification; both tumors were positive for HER3 protein expression.

**Table 2:** Distribution of markers in the total cohort and in HER2-positive and HER2-negative patients.

		HER2 status		
		Total	Negative	Positive
**Gene amplification (FISH)**	**EGFR** (gene/CEN ratio)	1.0 (0.5–4.0)	1.0 (0.5–4.0)	1.0 (0.6–2.0)
	Median (range)			
	**HER2** (gene/CEN ratio)	3.6 (0.6–26.6)	1.2 (0.6–2.0)	5.4 (1.1–26.6)
	Median (range)			
	**HER3** (gene/CEN ratio)	1.0 (0.4–2.0)	1.0 (0.7–1.5)	1.0 (0.4–2.0)
	Median (range)			
	**HER4** (gene/CEN ratio)	1.0 (0.6–1.4)	1.0 (0.7–1.3)	1.0 (0.6–1.4)
	Median (range)			
**Copy number variations (CNVs)**	**EGFR**			
	No gain	143 (91.7%)	54 (90.0%)	89 (92.7%)
	Gain	13 (8.3%)	6 (10.0%)	7 (7.3%)
	**HER2**			
	No gain	59 (36.0%)	48 (78.7%)	11 (10.7%)
	Low gain	63 (38.4%)	11 (18.0%)	52 (50.5%)
	High gain	42 (25.6%)	2 (3.3%)	40 (38.8%)
	**HER3**			
	No gain	137 (91.3%)	50 (87.7%)	87 (93.5%)
	Gain	13 (8.7%)	7 (12.3%)	6 (6.5%)
**Messenger RNA expression**	**EGFR**	37.1 (28.3–40.6)	36.7 (28.3–40.6)	37.3 (28.7–40.1)
	Median (range)			
	**HER2**	39.7 (27.0–44.4)	37.3 (27.0–40.2)	41.1 (37.0–44.4)
	Median (range)			
	**HER3**	41.3 (36.5–44.1)	41.1 (38.8–44.1)	41.3 (36.5–43.8)
	Median (range)			
	**HER4**	36.8 (25.5–40.4)	37.5 (27.0–40.4)	35.7 (25.5–40.3)
	Median (range)			
**Protein expression**	**EGFR**			
	Negative	149 (88.2%)	59 (89.6%)	90 (87.4%)
	Positive	20 (11.8%)	7 (10.4%)	13 (12.6%)
	**pHER2**^**Tyr1221/1222**^			
	Negative	117 (70.1%)	51 (77.3%)	66 (65.3%)
	Positive	50 (29.9%)	15 (22.7%)	35 (34.7%)
	**pHER2**^**Tyr877**^			
	Negative	135 (81.8%)	55 (84.6%)	80 (80.0%)
	Positive	30 (18.2%)	10 (15.4%)	20 (20.0%)
	**HER3**			
	Negative	49 (31.6%)	21 (33.3%)	28 (30.4%)
	Positive	106 (67.7%)	42 (66.6%)	64 (69.6%)
	**HER4**			
	Negative	36 (23.2%)	17 (27.0%)	19 (20.7%)
	Positive	119 (76.8%)	46 (73.0%)	73 (79.3%)

No significant differences were detected in the protein expression of EGFR, pHER2^Tyr1221/1222^, pHER2^Tyr877^, HER3 and HER4 between HER2-positive and HER2-negative patients (chi-square, p = 0.69, p = 0.10, p = 0.45, p = 0.70 and p = 0.36, respectively). Similarly, no significant differences were observed between HER2-positive and HER2-negative patients in terms of *EGFR* and *HER3* CNs (p = 0.55 and p = 0.22, respectively), while *HER2* CNs were found to be significantly different between HER2-positive and HER2-negative patients (Fisher’s exact test, p<0.001). Statistically significant differences between HER2-positive and HER2-negative participants were also observed in terms of HER4 and HER2 mRNA expression (Wilcoxon rank-sum, p = 0.025 and p<0.001, respectively), with HER2-positive patients presenting higher expression of HER2 mRNA and lower expression of HER4 mRNA. ER, PgR, Ki67, PTEN and *PIK3CA* data were presented in detail in two previous publications [8, 9]. The frequency of protein expression and CNVs of the markers of interest did not differ between *de novo* MBC and R-MBC patients, while HER3 mRNA expression (using the lower quartile as a cut-off) was more frequently observed in relapsed MBC patients (chi-square, p = 0.016).

Low EGFR mRNA expression (using the median value as a cut-off) was significantly associated with negative EGFR protein expression and positive HER3 protein expression (Fisher’s exact test, p = 0.012 and p = 0.049, respectively), while high HER3 mRNA expression (using the median value as a cut-off) was significantly associated with negative EGFR protein expression (p = 0.017). Negative EGFR protein expression was also found to be significantly associated with high HER4 mRNA expression (p<0.001), while HER4 mRNA expression (using the median value as a cut-off) was also associated with positive HER4 protein expression (p = 0.035). Associations between protein and mRNA expression of the markers are depicted in S1 Table.

## Association of HER family receptors with clinicopathological parameters

High HER2 mRNA expression (using the lower quartile as a cut-off) was significantly associated with visceral metastases (chi-square, p = 0.042). Positive HER3 protein expression was associated with positive ER/PgR status (p = 0.045), while positive ER/PgR status was also associated with negative EGFR protein expression (p<0.001). No significant associations were observed between subtypes and the expression of the examined markers. The association between HER family markers and PTEN protein status, *PIK3CA* mutations, as well as the combined PTEN/*PIK3CA* status was also examined. PTEN loss was associated with positive HER3 protein expression (chi-square, p = 0.038), while wild-type PIK3CA was associated with *HER2* low gain (p = 0.013). No further associations were observed between PTEN, *PIK3CA* or the combined PTEN/*PIK3CA* status and the examined markers.

## Association of markers with clinical outcome

**Fig 4:**
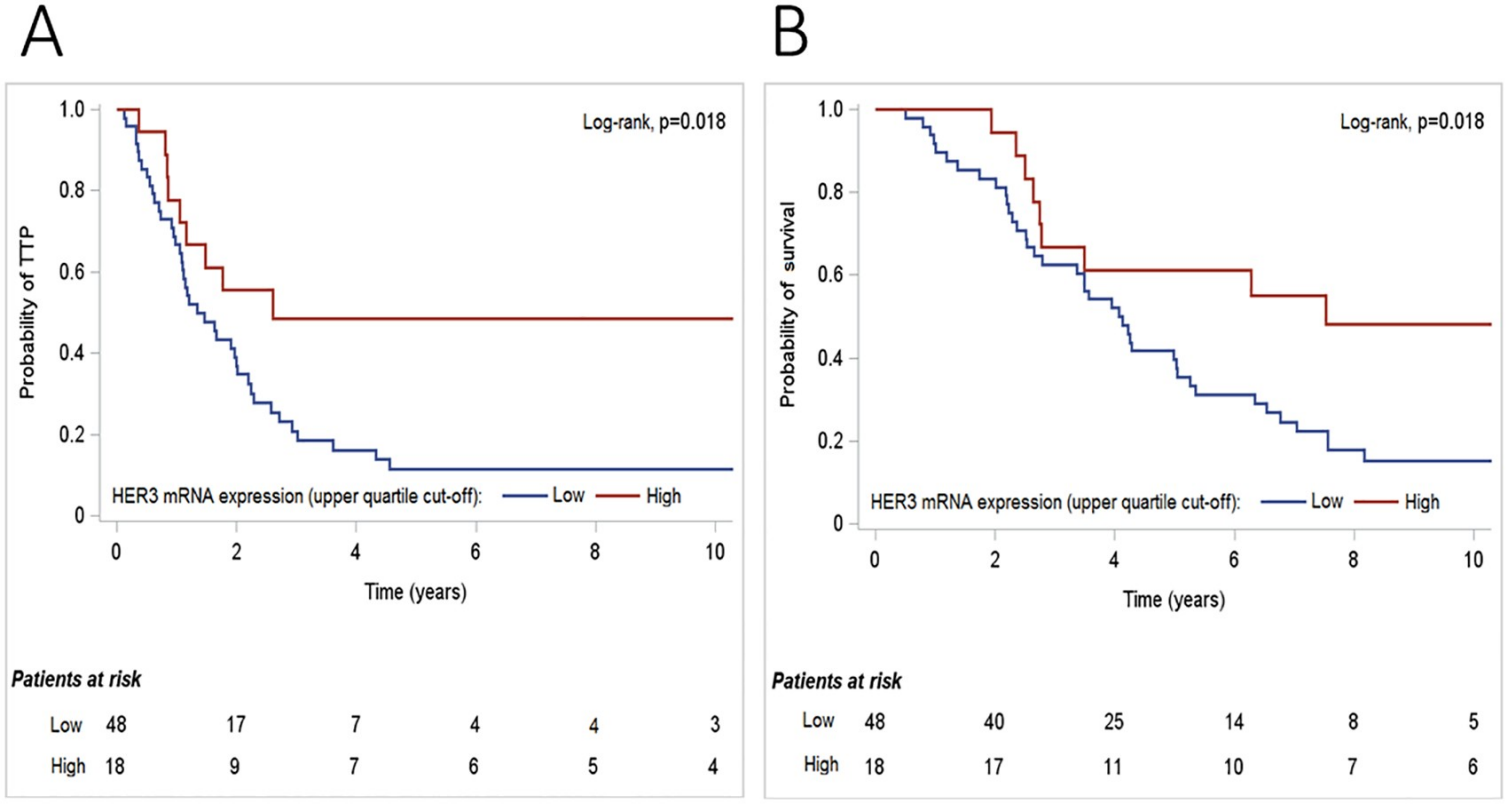
Kaplan-Meier curves in terms of (A) time to progression (TTP) and (B) survival, according to HER3 mRNA expression, in HER2-positive patients treated with first-line trastuzumab.

In the univariate analysis, with respect to TTP in HER2-positive patients, high HER3 mRNA expression (using the upper quartile as a cut-off) was significantly associated with lower risk for disease progression (HR = 0.43, 95% CI 0.21–0.88, Wald’s p = 0.022) (Fig 4).

In HER2-negative patients, *EGFR* copy gain and positive EGFR protein expression were associated with significantly higher risk for progression (HR = 3.53, 95% CI 1.19–10.50, p = 0.023 and HR = 3.37, 95% CI 1.12–10.17, p = 0.031, respectively). In contrast, positive HER3 protein expression was associated with lower risk for disease progression (HR = 0.43, 95% CI 0.22–0.84, p = 0.014). In the multivariate analyses, only *EGFR* copy gain retained its prognostic significance for TTP in the HER2-negative population (HR = 3.96, 95% CI 1.29–12.16, p = 0.016) (Table 3). However, it should be noted that the power of our study is limited by the small number of patients with available EGFR CNV and EGFR IHC data and therefore these results should be interpreted with caution since they need to be further validated in larger cohorts. The adjusted hazard ratio of HER2-negative patients with positive HER3 protein expression was 0.61 (95% CI 0.26–1.41), while statistical significance was not reached. High HER3 mRNA expression retained its favorable prognostic significance for TTP in the HER2-positive subgroup (HR = 0.47, 95% CI 0.23–0.99, p = 0.048).

**Table 3:** Results of the univariate and stratified multivariate Cox-regression analyses.

	Univariate				Multivariate			
Variable	N patiens / N events	HR	95% CI	p-value	N patiens/ N events	HR	95% CI	p-value
*HER2-positive*								
***TTP***								
**HER3 mRNA expression** High vs. low (upper quartile cut-off)	18 vs. 48 / 9 vs. 41	0.43	0.21–0.88	**0.022**	18 vs. 48 / 9 vs. 41	0.47	0.23–0.99	**0.048**
***Survival***								
**HER3 mRNA expression** High vs. low (upper quartile cut-off)	18 vs. 48 / 9 vs. 41	0.43	0.21–0.88	**0.021**	18 vs. 48 / 9 vs. 41	0.51	0.24–1.07	0.073
*HER2-negative patients*								
***TTP***								
**EGFR CNVs** Gain vs. no gain	4 vs. 44 / 4 vs. 37	3.53	1.19–10.50	**0.023**	4 vs. 43 / 4 vs. 37	3.96	1.29–12.16	**0.016**
**EGFR protein expression** Positive vs. negative	4 vs. 45 / 4 vs. 40	3.37	1.12–10.17	**0.031**	4 vs. 45 / 4 vs. 40	0.80	0.10–6.31	0.83
**HER3 protein expression** Positive vs. negative	30 vs. 16 / 23 vs. 16	0.43	0.22–0.84	**0.014**	29 vs. 16 / 23 vs. 16	0.61	0.26–1.41	0.25

N, number; HR, hazard ratio; CI, confidence interval.

In terms of survival, in the univariate analyses, high HER3 mRNA expression (using the upper quartile as a cut-off) was associated with lower risk for death in the HER2-positive population (HR = 0.43, 95% CI 0.21–0.88, p = 0.021), while none of the examined markers reached any significance for survival in the HER2-negative subgroup. In the multivariate analysis, high HER3 mRNA expression was marginally significantly associated with improved survival in HER2-positive patients (HR = 0.51, 95% CI 0.24–1.07, p = 0.073) (Table 3).

## Discussion

In the current study, we performed a complete analysis of gene amplification, copy number variations, transcriptional profiling and protein expression of all four HER family receptors, in a series of patients with MBC, treated with trastuzumab-based regimens. To the best of our knowledge, the current study may be the first to evaluate the role of HER family members, in the outcome of trastuzumab-treated patients with MBC, in such a comprehensive manner. Most of the available studies have assessed protein expression and/or gene amplification of specific HER family receptors in relatively small numbers of patients. Consequently, the clinical outcome of MBC with regard to HER family members expression as a whole panel remains largely undisclosed.

In addition, the present study includes the investigation of a HER2-negative, trastuzumab-treated subgroup. These patients had similar TTP and survival in comparison to the HER2-positive cases, probably due to the impact of trastuzumab on the prognosis of HER2-positive patients. The evaluation of this HER2-negative sub-population is considered important, given data suggesting that adjuvant trastuzumab might also be effective in HER2-negative tumors [27].

Considerable discordance rates between central and local evaluation of HER2 have also been reported in other studies [28]. Prospective analyses from two trastuzumab adjuvant trials have reported substantial discordance rates, as well. In the NSABP B-31 study, central review of the first 104 cases enrolled in the trial based on IHC results revealed that 18% of the community-based assays could not be confirmed centrally [29]. Similarly, tumor samples from the first 119 patients enrolled in the N9831 study were centrally tested. It was of interest that only 74% of these tumors were found to be 3+ by HercepTest and only 66% had HER2 gene amplification [30]. Since established treatment guidelines and standardization of techniques for HER2 status determination preclude the administration of trastuzumab in HER2-negative patients, our study provides a good opportunity to evaluate such a population.

In the present study, we demonstrated a negative prognostic value of *EGFR* copy gain for TTP in the HER2-negative subgroup. In a previous study [31], we investigated the potential prognostic value of the transcriptional profiling of all four HER family genes in patients with high-risk early breast cancer. We demonstrated a reduced overall survival (OS) in patients with increased EGFR mRNA expression. Moreover, in a validation study conducted by our group in patients with high-risk early breast cancer [32], a negative prognostic value of EGFR protein expression was demonstrated for OS and disease-free survival (DFS) in the multivariate analysis. In general, EGFR is considered to be a negative prognostic factor in patients with breast cancer and such an association has been shown in ours, as well as in other studies [33–36].

Moreover, in the univariate analysis of our study, positive EGFR protein expression was associated with increased risk for disease progression in the HER2-negative population. Clinical data regarding the association between EGFR expression and trastuzumab activity remain largely inconclusive. A recent study that evaluated the association between the quantitative immunofluorescence-based assessment of EGFR expression and clinical outcome in the North Central Cancer Treatment Group (NCCTG) N9831 trial showed that high protein expression of EGFR was associated with decreased benefit from adjuvant trastuzumab, given concurrently with chemotherapy [37]. In addition, a recent study reported that EGFR protein overexpression is a poor prognostic marker, as well as a negative predictive factor for trastuzumab treatment, in patients with HER2-positive primary breast cancer [38]. Moreover, in another study including a cohort of 47 HER2-positive patients with metastatic breast cancer treated with trastuzumab, EGFR protein expression was correlated with worse OS [39]. Furthermore, a marginally significant negative association between protein expression of EGFR using IHC and pathologic complete response (pCR) has been observed in 44 HER2-positive patients treated with trastuzumab-containing neo-adjuvant chemotherapy [40]. In contrast, in another study including 45 patients with HER2-positive MBC treated with trastuzumab, EGFR status evaluated by IHC was not associated with response to trastuzumab, TTP or OS [17].

Regarding the transcriptional profiling of the HER2 gene, no significant relationship between HER2 mRNA expression and TTP or survival was demonstrated in the present analysis. However, a recent study conducted in the same cohort of patients using a new method of mRNA in situ hybridization (marketed as RNAscope) showed that measurement of HER2 mRNA expression with this novel method has a predictive value for trastuzumab-based chemotherapy, in MBC [41]. The discrepancy in these results highlights the importance of HER2 testing optimization.

With respect to the HER3 receptor, the univariate analysis of the present study showed that it might represent a positive prognostic factor for TTP and/or survival in both HER2-positive and HER2-negative patients. In the multivariate analyses, high HER3 mRNA expression retained its favorable prognostic significance for TTP in the HER2-positive subgroup. Moreover, high HER3 mRNA expression was marginally significantly associated with improved survival in HER2-positive patients. Although it is considered that HER3 may play a role in mediating resistance to trastuzumab, the available clinical data are not always in agreement. A recently published study in patients with MBC treated with trastuzumab-based therapy showed that within the subgroup of cases that overexpressed HER2, high levels of HER3 and/or p95 protein expression were significantly associated with poor clinical outcome [42]. In contrast, another publication demonstrated that HER3 status by immunohistochemistry was not significantly correlated with outcome in HER2-positive MBC patients receiving trastuzumab-based therapy [43]. In another study from our group including patients with early breast cancer [31], we found that HER3 mRNA expression was associated with longer OS. The existing data concerning the prognostic significance of HER3 in breast cancer patients are not conclusive. Even though a negative prognostic role of HER3 is suggested through a considerable number of publications [35, 44, 45], other studies indicate a positive prognostic value [33, 46, 47]. Although the reason for such inconsistencies among the literature data is not clear, a possible explanation could be related to the sub-cellular distribution of the HER3 receptor, which may be affected by the expression of HER3 ligands [48]. Hypothetically, these factors may influence the prognostic ability of HER3, potentially affecting the biological activities of the receptor [49].

Regarding HER4, no significant associations were observed in the current study. A previous study that retrospectively investigated the expression of all HER receptors using IHC and FISH, in a cohort of 48 patients with advanced breast cancer treated with trastuzumab, reported a significant positive impact of HER4 expression on survival [50]. In contrast to this observation, we did not find any significant associations between HER4 (mRNA or protein expression) and clinical outcome in the HER2-positive patients receiving trastuzumab-based treatment. In our previously reported studies in early breast cancer patients receiving adjuvant chemotherapy [31, 32], a positive association of HER4 mRNA expression with DFS and OS was observed. Similarly, other studies have also demonstrated a positive prognostic ability of HER4 in patients with breast cancer, both at the mRNA and the protein level [33, 35, 51].

## Conclusions

The present study suggests that high HER3 mRNA expression is of favorable prognostic significance for TTP and survival in HER2-positive MBC patients treated with first-line trastuzumab. In addition, *EGFR* copy gain and EGFR protein expression appear to constitute negative prognostic factors for TTP in patients with HER2-negative MBC, while HER3 protein expression is a favorable prognostic factor. It should be noted however that the latter findings are limited by the small number of patients and should therefore be interpreted with caution. The current analysis is fraught with the drawbacks of all retrospective studies particularly those that arise from a non-trial patient population and therefore, our findings should be viewed as hypothesis generating rather than definitive.

## Supporting information

S1 Table Associations of EGFR, HER2, HER3 and HER4 protein expression with mRNA expression. (DOCX)
