# Supplementary material for: Evaluation of the prognostic value of all four HER family receptors in patients with metastatic breast cancer treated with trastuzumab: A Hellenic Cooperative Oncology Group (HeCOG) study
Source: PLoS One. 2018 Dec 6;13(12):e0207707. doi: 10.1371/journal.pone.0207707 (PMC6283464; doi:10.1371/journal.pone.0207707)
Supplement: S1 Table — (DOCX) [file pone.0207707.s001.docx]

S1 Table. Associations of EGFR, HER2, HER3 and HER4 protein expression with mRNA expression.

|  |  | **mRNA expression** | | | | | | | | | | | |
| --- | --- | --- | --- | --- | --- | --- | --- | --- | --- | --- | --- | --- | --- |
| **Protein expression** |  | EGFR (median value as cut-off) | | | HER2 (median value as cut-off) | | | HER3 (median value as cut-off) | | | HER4 (median value as cut-off) | | |
|  |  | Low | High | P-value | Low | High | P-value | Low | High | P-value | Low | High | P-value |
|  | | | | |  | | | | | |  | | |
| EGFR | Negative | 42 (95.5) | 37 (77.1) | **0.012** | 44 (88.0) | 41 (82.0) | 0.40 | 37 (77.1) | 47 (94.0) | **0.017** | 33 (68.8) | 47 (100.0) | **<0.001** |
|  | Positive | 2 (4.5) | 11 (22.9) |  | 6 (12.0) | 9 (18.0) |  | 11 (22.9) | 3 (6.0) |  | 15 (31.3) | 0 (0.0) |  |
|  | | | | |  | | | | | |  | | |
| pHER2^Tyr1221/1222^ | Negative | 30 (68.2) | 33 (70.2) | 0.83 | 37 (75.5) | 30 (61.2) | 0.13 | 32 (68.1) | 33 (67.3) | 0.94 | 35 (72.9) | 30 (65.2) | 0.42 |
|  | Positive | 14 (31.8) | 14 (29.8) |  | 12 (24.5) | 19 (38.8) |  | 15 (31.9) | 16 (32.7) |  | 13 (27.1) | 16 (34.8) |  |
|  | | | | |  | | | | | |  | | |
| pHER2^Tyr877^ | Negative | 38 (88.4) | 33 (73.3) | 0.074 | 41 (85.4) | 38 (79.2) | 0.42 | 36 (80.0) | 41 (83.7) | 0.64 | 37 (80.4) | 37 (82.2) | 0.83 |
|  | Positive | 5 (11.6) | 12 (26.7) |  | 7 (14.6) | 10 (20.8) |  | 9 (20.0) | 8 (16.3) |  | 9 (19.6) | 8 (17.8) |  |
|  | | | | |  | | | | | |  | | |
| HER3 | Negative | 5 (12.2) | 12 (30.0) | **0.049** | 7 (16.3) | 9 (20.9) | 0.58 | 9 (22.5) | 5 (11.6) | 0.19 | 8 (21.1) | 10 (21.3) | 0.98 |
|  | Positive | 36 (87.8) | 28 (70.0) |  | 36 (83.7) | 34 (79.1) |  | 31 (77.5) | 38 (88.4) |  | 30 (78.9) | 37 (78.7) |  |
|  | | | | |  | | | | | |  | | |
| HER4 | Negative | 5 (12.2) | 7 (17.5) | 0.50 | 7 (16.3) | 5 (11.6) | 0.53 | 6 (15.0) | 6 (14.0) | 0.89 | 2 (5.3) | 10 (21.3) | **0.035** |
|  | Positive | 36 (87.8) | 33 (82.5) |  | 36 (83.7) | 38 (88.4) |  | 34 (85.0) | 37 (86.0) |  | 36 (94.7) | 37 (78.7) |  |
